# Successful every-other-day liothyronine therapy for severe resistance to thyroid hormone beta with a novel *THRB* mutation; case report

**DOI:** 10.1186/s12902-015-0081-7

**Published:** 2016-01-12

**Authors:** Yoshihiro Maruo, Asami Mori, Yoriko Morioka, Chihiro Sawai, Yu Mimura, Katsuyuki Matui, Yoshihiro Takeuchi

**Affiliations:** Department of Pediatrics, Shiga University of Medical Science, Tsukinowa, Seta, Otsu, 520-2192 Japan

**Keywords:** Congenital hypothyroidism, Resistance to thyroid hormone, Thyroid hormone receptor β, Liothyronine

## Abstract

**Background:**

Resistance to thyroid hormone beta (RTHβ) is a rare and usually dominantly inherited syndrome caused by mutations of the thyroid hormone receptor β gene (*THRB*). In severe cases, it is rarely challenging to control manifestations using daily therapeutic replacement of thyroid hormone.

**Case presentation:**

The present case study concerns an 8-year-old Japanese girl with a severe phenotype of RTH (TSH, fT3, and fT4 were 34.0 mU/L, >25.0 pg/mL and, >8.0 ng/dL, respectively), caused by a novel heterozygous frameshift mutation in exon 10 of the thyroid hormone receptor beta gene (*THRB*), c.1347-1357 del actcttccccc : p.E449DfsX11. RTH was detected at the neonatal screening program. At 4 years of age, the patient continued to suffer from mental retardation, hyperactivity, insomnia, and reduced resting energy expenditure (REE), despite daily thyroxine (L-T4) therapy. Every-other-day high-dose liothyronine (L-T3) therapy improved her symptoms and increased her REE, without thyrotoxicosis.

**Conclusion:**

In a case of severe RTH, every-other-day L-T3 administration enhanced REE and psychomotor development, without promoting symptoms of thyrotoxicosis. Every-other-day L-T3 administration may be an effective strategy for the treatment of severe RTH.

## Background

Resistance to thyroid hormone beta (RTHβ, #MIM 188570) is a rare syndrome characterized by reduced responsiveness of some target tissues to thyroid hormone. RTHβ is usually inherited as an autosomal dominant trait, and is primarily caused by a mutation of the thyroid hormone receptor β gene (*THRB*) [1]. *THRB* is located on chromosome 17 and consists of 10 exons. More than 100 mutations have been reported; with the exception of 1 family, all mutations are located on exons 7–10 [2]. The mutant receptors are able to interfere with the function of the wild-type receptor, known as a dominant-negative effect. As such, heterozygous carriers often suffer from RTHβ. The symptoms of RTHβ are variable depending on the type of mutation. Many patients show a mild phenotype, and typically, treatment is not required. Heterozygous mutations resulting in truncated THRB cause a severe phenotype. Specifically, THRB lacking the last 20–28 amino acid residues causes mental retardation, visual and auditory deficits, and short stature [3]. Patients with biallelic mutations of *THRB* also develop a severe phenotype [4, 5]. Most children with RTHβ experience attention-deficit hyperactivity disorder (ADHD) [6]. In children with RTHβ and ADHD, particularly those who exhibit hyperactivity, liothyronine (L-T3), in supra-physiological doses, may be beneficial in reducing hyperactivity and impulsivity [7].

We report a severe case of RTHβ in an 8-year-old girl, in whom we detected a novel heterozygous deletion mutation of *THRB*, which generates a truncated protein. The patient experienced mental retardation, hyperactivity, and insomnia, despite treatment with thyroxine (L-T4) therapy. Using every-other-day L-T3 therapy in large doses improved her symptoms without thyrotoxicosis.

## Case presentation

The ethics committee of the Shiga University of Medical Science approved the study. The patient is the first child of the family, born to non-consanguineous parents without a family history of thyroid disease. Informed consent was obtained from the patient’s parents.

**Fig. 1:**
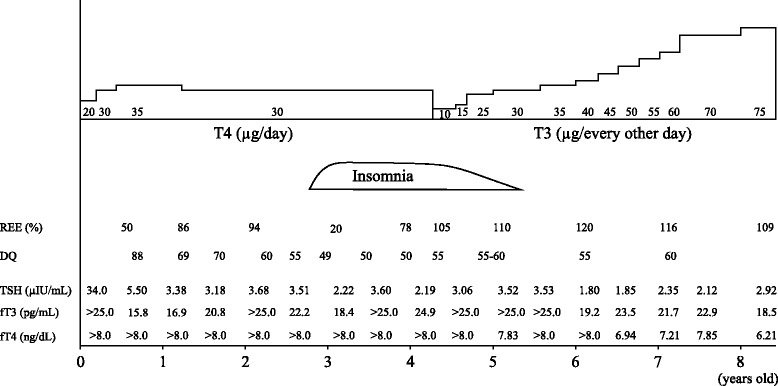
Clinical course of the patient. REE; resting energy expenditure, DQ; developmental quotient

At 20 days of age, the patient’s serum TSH level increased to 198 μU/mL at neonatal screening. At 26 days of age, she visited our outpatient clinic exhibiting failure to thrive, persistent jaundice, and umbilical herniation. The patient’s serum TSH, fT3, fT4, total T3, total T4, and TG values were 34.0 mU/L, >25.0 pg/mL, >8.0 ng/dL, 6.20 ng/mL, >24.9 μg/dL, and >800 ng/mL, respectively. She had no signs of heat intolerance, tachycardia, or hyperactivity. Thyroid gland enlargement and delayed bone maturation were noted. Pituitary size, as assessed with magnetic resonance imaging, indicated slight anterior lobe hyperplasia. TRH stimulation tests showed an excessive response (peak TSH: 92 μU/ml). The resting energy expenditure (REE), which was measured using AE300S (Minato Medical Science, Japan) by indirect calorimetry [REE (kcal/day) = 5.616 x VO_2_ (ml/min) + 1.584 x VCO_2_ (ml/min)] [8], was one-third of the normal values typical for her age. No anti-thyroid antibodies were detected. Based on these results, the patient was diagnosed with RTH. The patient was treated with L-T4 replacement starting at 2 weeks of age (7.5 μg/kg/day). After initiating replacement, TSH levels reduced to normal and her weight gain and REE improved (Fig. 1).

At 2 years of age, the patient showed normal growth (height and weight). However, she developed mental retardation [developmental quotient (DQ): 70–60 (postural-motor region 52, cognitive-adaptive region 63, language-social region 68) (the Kyoto Scale of Psychological Development new edition 2001)] and a learning disability associated with ADHD. At 4 years of age, the patient’s DQ reduced to 50 and she showed symptoms of hyperactivity and insomnia. Her bone age was delayed about 1.5 years behind her chronological age, and REE decreased to 78 % of 4-years-girl. To enhance the patient’s psychomotor development and REE, we chose to increase the L-T4 dose. However, considering the consistently high fT3, secondary to L-T4 therapy which could worsen insomnia, hyperactivity, and ADHD, the therapy was changed to single every-other-day large doses of L-T3 (75 μg). After start of L-T3 every-other-day replacement in one year, her DQ increased to 60 (Fig. 1). The patient’s insomnia and hyperactivity also improved. At the time of publication, the patient is 8 years, 11 months old, with a height of 131.6 cm (+0.28 SD). Her recent TSH, fT3, fT4, and TG were 2.92 μIU/mL, 18.5 pg/mL, 6.21 ng/dL, and 85.9 ng/mL, respectively. REE was 109.3 % of the predicted values for an 8-year-old girl (Fig. 1).

### Laboratory testing and sequence analysis

TSH was measured using a fluorescent enzyme immunoassay method (TOSHO, Tokyo, Japan). fT4 and fT3 were measured with a fluorescent enzyme immunoassay (TOSHO, Tokyo, Japan). Thyroglobulin was measured by an immunoradiometric assay (EIKEN CHEMICAL, Tokyo, Japan).

Blood samples were obtained from the patient and her parents. Genomic DNA was isolated from leukocytes by the standard protocol. *THRB* exons were amplified by polymerase chain reaction using a previously reported protocol [9]. The sequences of the amplified DNA fragments were determined directly using a BigDye® Terminators v1.1 Cycle Sequencing Kit (Applied Biosystems, CA) and an ABI PRISM 3130xI Genetic Analyzer (Applied Biosystems, CA).

Homology models of wild type and mutant THRB were predicted using a molecular operating environment (MOE; Chemical Computing Group Inc., Montreal, Quebec, Canada). The query amino acid sequence of human THRB came from GenPret (Accession number P10828). Thyroid receptor alpha (PDB code 1NAVA) was selected as a template structure by alignment of amino acid sequences in the MOE protein database [10].

## Results

### Gene analysis

**Fig. 2:**
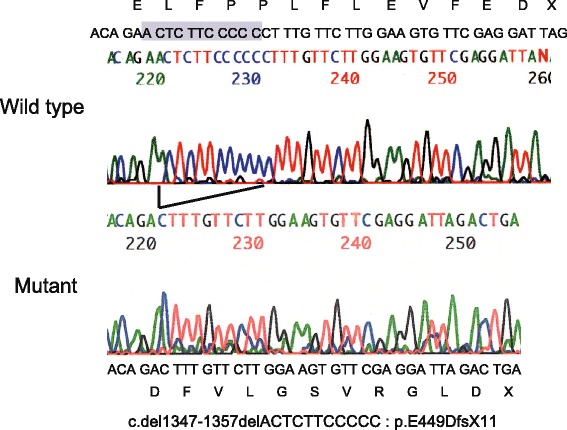
THRB nucleotide sequences amplified from the genomic DNA of the patient. The mutation, a deletion of “actcttccccc” at position 1347_1357 in THRB cDNA, changed the amino acid sequence from 449–461 “ELFPPLFLEVFED” to 449–459 “DFVLGSVRGLD” and made two amino acids in the T3 binding domain shorter, when compared with wild type THRB

We detected a novel deletion mutation in exon 10 of *THRB* (Fig. 2). The 11 bases of nucleotide sequence, “actcttccccc” at 1347_1357 were deleted. This deletion leads to a frameshift, producing a stop at codon 459: c.1347_1357 del actcttccccc (p.E449DfsX11) (Fig. 1). Moreover, this mutation introduces changes in the amino acid sequence from 449–461 “ELFPPLFLEVFED” to 449–459 “DFVLGSVRGLD”, and causes two amino acids in the T3 binding domain to be shorter, when compared with wild type THRB. The patient’s parents did not show any mutations on *THRB*.

### Comparison of the conformation of wild type with p.E449DfsX11 THRB

**Fig. 3:**
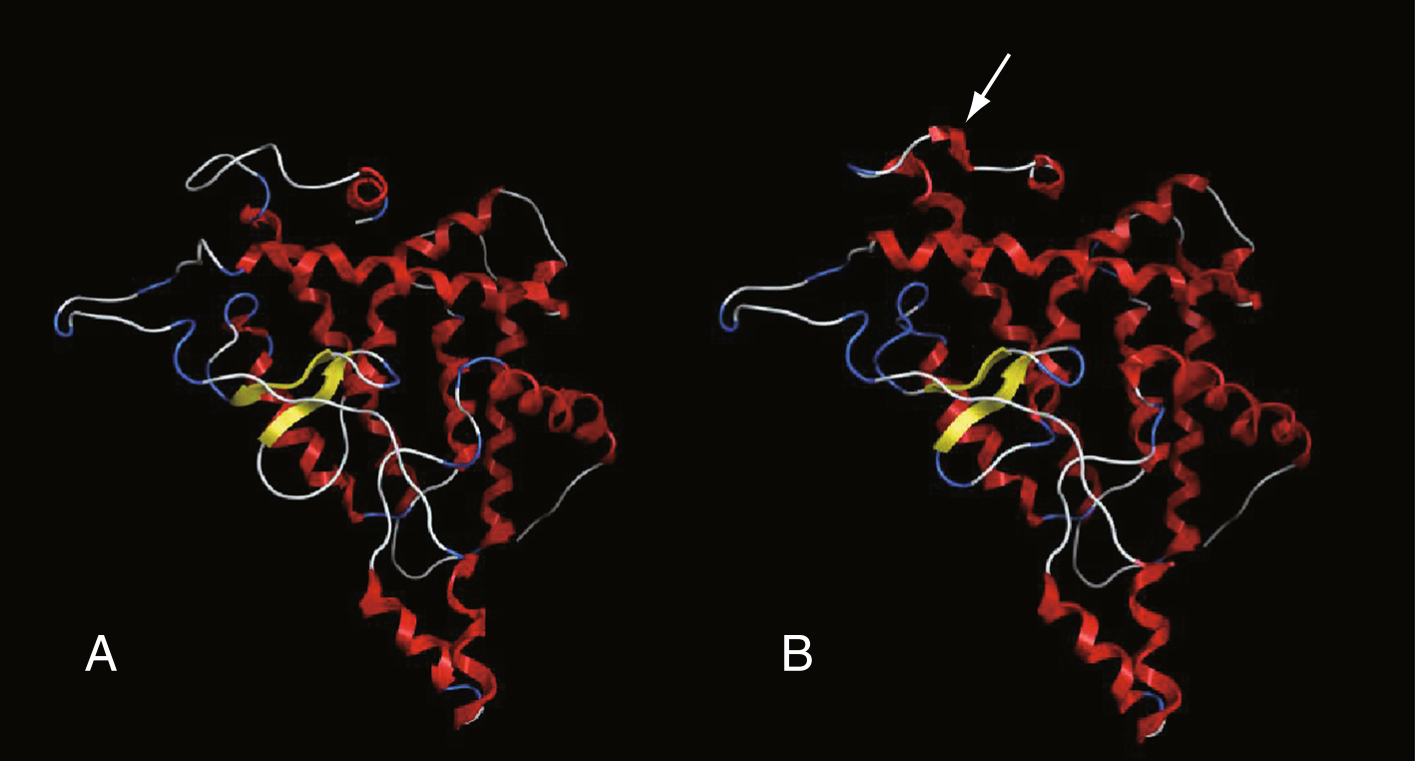
Ribbon diagrams of the predicted THRB structure from wild type (**a**) and p.E449DfsX11 mutant protein (**b**). The figures were produced with homology-based modeling using MOE. Red ribbons represent α-helices, and yellow arrows indicate the β-sheet. The mutated THRB has one different α-helix (amino acid number 450–451) (arrows)

We used MOE to predict model 3D structures of wild type and p.E449DfsX11 THRB. The p.E449DfsX11 THRB has one different α-helix (amino acid number 450–451) at the C-terminal (Fig. 3). The deletion mutation with amino acid replacement causes significant changes to the tertiary structure of THRB.

## Discussion

Usually, patients with RTH are euthyroid and do not require treatment with thyroid hormone. However, in severe cases, to prevent goiter development, daily L-T4 or L-T3 therapeutic replacement is necessary. It is rarely challenging to control manifestations by using daily therapeutic replacement of L-T4 or L-T3. Every-other-day L-T3 replacement has shown to be effective for treating uncontrolled large goiters in patients with severe RTH, without symptoms of thyrotoxicosis or severely elevated TSH [11, 12]. However, effects on psychological and mental improvement have not been reported. Supra-physiological doses of L-T3 can reduce hyperactivity and impulsivity [7]. However, for patients with severe RTH, such as the present case, extreme elevations in T3 level might worsen psychological and mental development. Daily L-T4 supplementation also induces consistently high T3 levels, which may worsen insomnia, hyperactivity, and ADHD. To suppress TSH levels and prevent consistently high T3 levels, every-other-day L-T3 replacement might be an effective. For RTHβ, treatment of β blocker and 3,5,3'-triiodothyroacetic acid (TRIAC) was also considered as well as L-thyroxine if necessary. Usually REE of patients with RTHβ increases. However decline of REE in our patient suggested that she is in state of hypothyroidism physically. The treatment with L-T3 was chosen. Her mutated THRB might strongly interfere function of the wild THRB. Severe reduction of THRB function might induce low REE.

The novel mutation, c.1347-1357 del actcttccccc, generates a premature stop codon at 459 with an altered C-terminal amino acid sequence (p.E449DfsX11) (Fig. 1). These changes in the 3-dimensional structure of C-terminal might cause severe RTH (Fig. 2). These changes might cause severe RTH in patient with the heterozygous mutation (p.E449DfsX11).

## Conclusions

For severe RTH, every-other-day L-T3 therapy may be an effective therapy to improve REE and psychomotor development, without inducing symptoms of thyrotoxicosis.

## Consent

The parents of the patient have given their consent for the Case reports to be published.

## Abbreviations

RTH: resistance to thyroid hormone
THRB: thyroid hormone receptor β
L-T3: liothyronine
L-T4: thyroxine
TG: thyroglobulin
